# Discrimination of Geographical Origin of Agricultural Products From Small-Scale Districts by Widely Targeted Metabolomics With a Case Study on Pinggu Peach

**DOI:** 10.3389/fnut.2022.891302

**Published:** 2022-05-24

**Authors:** Jie Zhao, An Li, Xinxin Jin, Gang Liang, Ligang Pan

**Affiliations:** ^1^Institute of Quality Standard and Testing Technology, Beijing Academy of Agriculture and Forestry Sciences, Beijing, China; ^2^Risk Assessment Lab for Agro-Products, Ministry of Agriculture, Beijing, China

**Keywords:** Analytical GREEnness metric approach, metabolic fingerprint, origin discrimination, peach, small-scale districts, widely targeted metabolomics

## Abstract

Geographical indications of agricultural products are characterized by high quality and regional attributes, while they are more likely to be counterfeited by similar products from nearby regions. Accurate discrimination of origin on small geographical scales is extremely important for geographical indications of agricultural products to avoid food fraud. In this study, a widely targeted metabolomics based on ultra-high-performance liquid chromatography–tandem mass spectrometry combined with multivariate statistical analysis was used to distinguish the geographical origin of Pinggu Peach of Beijing and its two surrounding areas in Heibei province (China). Orthogonal partial least squares-discriminant analysis (OPLS-DA) based on 159 identified metabolites showed significant separation from Pinggu and the other adjacent regions. The number of the most important discriminant variables (VIP value >1) was up to 62, which contributed to the differentiation model. The results demonstrated that the metabolic fingerprinting combined with OPLS-DA could be successfully implemented to differentiate the geographical origin of peach from small-scale origins, thus providing technical support to further ensure the authenticity of geographical indication products. The greenness of the developed method was assessed using the Analytical GREEnness Metric Approach and Software (ARGEE) tool. It was a relatively green analytical method with room for improvement.

## Introduction

Food fraud, motivated by economic profit, which includes adulteration, misbranding, false geographical origin, untrue agricultural production, and so on, is a growing worldwide concern among consumers, government agencies, policymakers, industry, and scientists in recent years (1). Food authenticity is not only related to the quality and safety of food but also an influencing factor for consumers' interests and the rebuilding of consumer confidence (2). Geo-origin is one of the most important aspects of food authenticity, consumers have the right to know the complete information of the food they are eating (3). However, the food trade globalization and the lack of reliable information during food production and processing present challenges to food trustworthiness. Thus, determining food origin becomes essential in food supply chain management.

Geographical indication (GI) is a sign used on products that have a specific geographical origin and possess qualities or a reputation that are due to that origin, which is defined by the World Intellectual Property Organization (4). GI is not only the local symbol but also a quality and reputation sign, which contains a huge economic and cultural value. Some GI products have even become a major force and industrial pillar for regional economic growth. However, with the higher retail price and higher financial benefit of GI products, the commercial fraud issue is usually more likely to occur (5). China is promoting the rural revitalization strategy to realize economic development and poverty reduction; therefore, the protection of good GI products is vital (6).

China, as one of the origins of peach, is the largest peach production country in the world. Pinggu, one district of Beijing, is the largest peach-producing region in China, known as the “Homeland of Chinese peach.” Pinggu District has also received the honorary titles “China's High-Quality Peach Production Base” and “Nationally Advanced District for Standardized Peach Production.” Pinggu Peach is the Chinese famous GI product known for its quality and flavor. It has been exported to more than 20 countries in Asia, Europe, and America. In addition, Pinggu Peach is one of China's 10 native products with the European Union protection (7); its brand value was estimated as more than 10 billion in 2019. However, the counterfeiting by very similar peach products from nearby regions is still happening in the market, which damages the interests of the consumers and producers and is not conducive to promoting the long-term development of the brand. How to accurately distinguish the producing areas of peaches in adjacent areas or in small-scale districts is the key to solve this problem.

So far, a significant number of analytical methods have been reported for identifying food geographical origins, such as isotopic analysis to trace the geographical origin of trout (8), amino acid carbon stable isotope fingerprinting to authenticate sea cucumber (9), multi-element analysis to determine the pork and tea (10, 11), combination of elemental and isotopic composition to discriminate Asian rice and green tea in China (12, 13), organic composition analysis to separate the geographical origins of *Lycium ruthenicum* Murray (14), and DNA barcoding or DNA fingerprinting to determine the geographical origin of fruits and mushrooms (15–17). These methods can well trace the geographical origins of multiple foods from a large geographical scope, such as different countries and even provinces. However, enough attention is not paid to distinguishing similar products from nearby places, which is more challenging. Also, appropriate technologies or methods have not been well developed and applied.

Omics-based technologies, such as metabolomics, related to the strength of massive molecular tools can help circumvent the limitations of traditional methodologies, and therefore are being developed for the authentication of a wide range of food commodities, such as organic carrot authentication (18), food ingredient authenticity (19), geographical origins of oranges and shrimps, wine and cheese origin discrimination (20–23), and fruit juice authenticity (24, 25). Widely targeted metabolomics analysis is a novel approach that integrates the advantages of nontargeted and targeted metabolites. Based on multiple reaction monitoring (MRM) and multiple ion monitoring, the aforementioned analysis is a sensitive and accurate method for simultaneously quantifying hundreds of known metabolites and nearly 1000 known and unknown metabolites (26). Meanwhile, Rychlik et al. estimated that the conservative number of all considered food metabolites could be 500,000; it is expected that the analysis of food metabolites may play an important role in food field (27). In recent years, widely targeted metabolomics analysis has been successfully applied to analyze the metabolic response, metabolic alterations, and metabolite accumulation in plants (28–30). Therefore, this study aimed to estimate the feasibility of authenticating peach origins from adjacent producing regions using the widely targeted metabolomics technique and provide a reliable geographical origin discrimination method for GI product protection.

## Materials and Methods

### Chemicals and Reagents

Chromatogram-class methanol and acetonitrile were supplied by Honeywell (NJ, USA), and formic acid was supplied by Sigma–Aldrich (MO, USA). Ultrapure water (18.3 MΩ cm) was obtained from a Milli-Q purification system (Merck Millipore, Darmstadt, Germany).

### Sample Collection and Treatment

**Figure 1:**
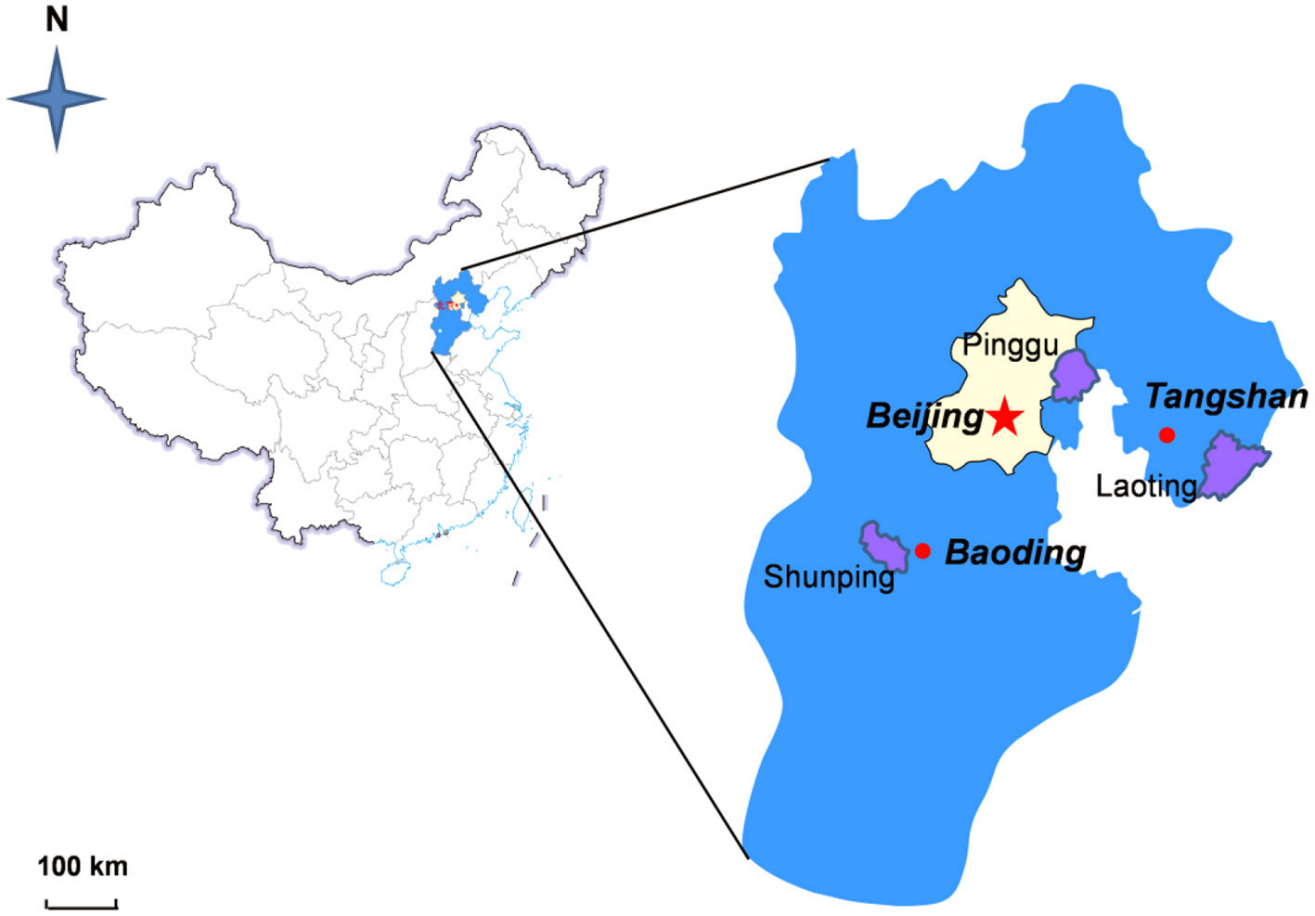
Geographical map of peach sampling sites from different regions in China and the specific localities of peach samples within Pinggu (Beijing, capital city), Shunping (Hebei province), and Laoting (Hebei province).

“Okubao” peach samples, which are a widely cultivated and popular variety, were collected from three adjacent and different ecotype regions in the harvest season. The regions were Pinggu Beijing (PG, the only large-concentrated contiguous mountain plain in North China), Laoting Hebei province (LT, belongs to the coastal plain), and Shunping Hebei province (SP, classified as the inland plain region). The geographical location map of peach samples is shown in Figure 1. A total of 24 representative peach samples with a medium size (diameter around 9 cm) and uniform maturity from the three areas were used for detecting metabolites. The samples were treated using a liquid-nitrogen flash freezer and saved at −80°C until use.

### Extraction of the Metabolites of Samples

Small pieces of samples were initially freeze-dried and then crushed with a mixer to extract the metabolites. Then, 100 mg powder of each sample was extracted with 1000 μL of methanol/water mixture (*v*:*v* = 3:1) with vortexing for 30 s and then treated ultrasonically for 15 min in an ice bath. The samples were shaken overnight and centrifuged at 10 000 g for 15 min at 4°C. Then, 500 μL of the supernatants were dried under gentle nitrogen flow. The residue of each sample was re-dissolved in 250 μL of methanol/water mixture (*v*:*v*=1:1) with vortexing for 30 s and sonicated at maximum frequency (40 kHz) continuously for 15 min in the ice bath. Afterward, the samples were centrifuged at 10 000 g for 15 min at 4°C (31). The resulting supernatant was taken for ultra-high-performance liquid chromatography–tandem mass spectrometry (UHPLC-MS/MS) analysis. The quality control (QC) sample was prepared by mixing an equal aliquot of every sample and injected five times to ensure the stability and repeatability of the system.

### Metabolite Acquisition and Identification

A widely targeted metabolomics analysis was conducted on a Waters ACQUITY UPLC (MA, USA) coupled with a Thermo Q Exactive Focus mass spectrometer (MA, USA) and a SCIEX 6500 Qtrap mass spectrometer (MA, USA) to evaluate the differences in metabolites among the three regions. The chromatographic separation was achieved on a Waters UHPLC HSS T3 column (2.1 mm × 100 mm, 1.8 μm) maintained at 40°C. The injection volume was 2 μL, and the flow rate was 300 μL/min. The mobile phase A was 0.1% formic acid in water, and the mobile phase B was acetonitrile. The solvent gradient was as follows: 0–2 min, 2% B; 2–11 min, 2–98% B; 11–13 min, 98% B; 13–15 min, 98%−2% B (32).

The QE Focus mass spectrometer (Thermo, MA, USA) was used to acquire high-resolution MS/MS spectrum data in an information-dependent acquisition mode. In this mode, the acquisition software (Xcalibur 4.1, Thermo, MA, USA) continuously evaluated the full scan survey MS data while collecting and triggering the acquisition of MS/MS spectra depending on preselected criteria. In each cycle, three precursor ions whose intensities were greater than 5000 were chosen for fragmentation at collision energy. Acquired mass range was divided into 70–300, 290–600, and 590–1100 with three injections. Elaectrospray Ionization source conditions were set as follows: spray voltage: +3500/−3500 V; capillary temperature: 350°C; sheath gas: 30; aux gas: 10; CE: 10, 30, and 50. A Qtrap 6500 (MA, USA) mass spectrometer was applied for metabolite quantification in the MRM mode. The ion source parameters were as follows: ion spray voltage: +5000/−4500 V; curtain gas: 35 psi; temperature: 500°C; ion source gas I: 55 psi; ion source gas II: 60 psi; declustering potential: ±100 V (33).

### Statistical Analysis

The raw MS data files were converted into the mzXML format using ProteoWizard software and pre-processed (peak detection, data mining, alignment, and normalization) using MAPS software (version 3.2) (34). The metabolites were identified based on the in-house MS2 database, Human Metabolome Database (HMDB), and Kyoto Encyclopedia of Genes and Genomes (KEGG) database with Skyline software. The multivariate statistical analysis, including principal component analysis (PCA) and orthogonal partial least squares-discriminant analysis (OPLS-DA), was performed on mass spectral data sets using SIMCA 14.1 software (Umetrics, Umeå, Sweden). Hierarchical cluster analysis (HCA) was performed with R software (www.r-project.org/). KEGG pathway analysis was used to annotate and enrich differential metabolites (35).

## Results

### Data Quality Assessment

**Figure 2:**
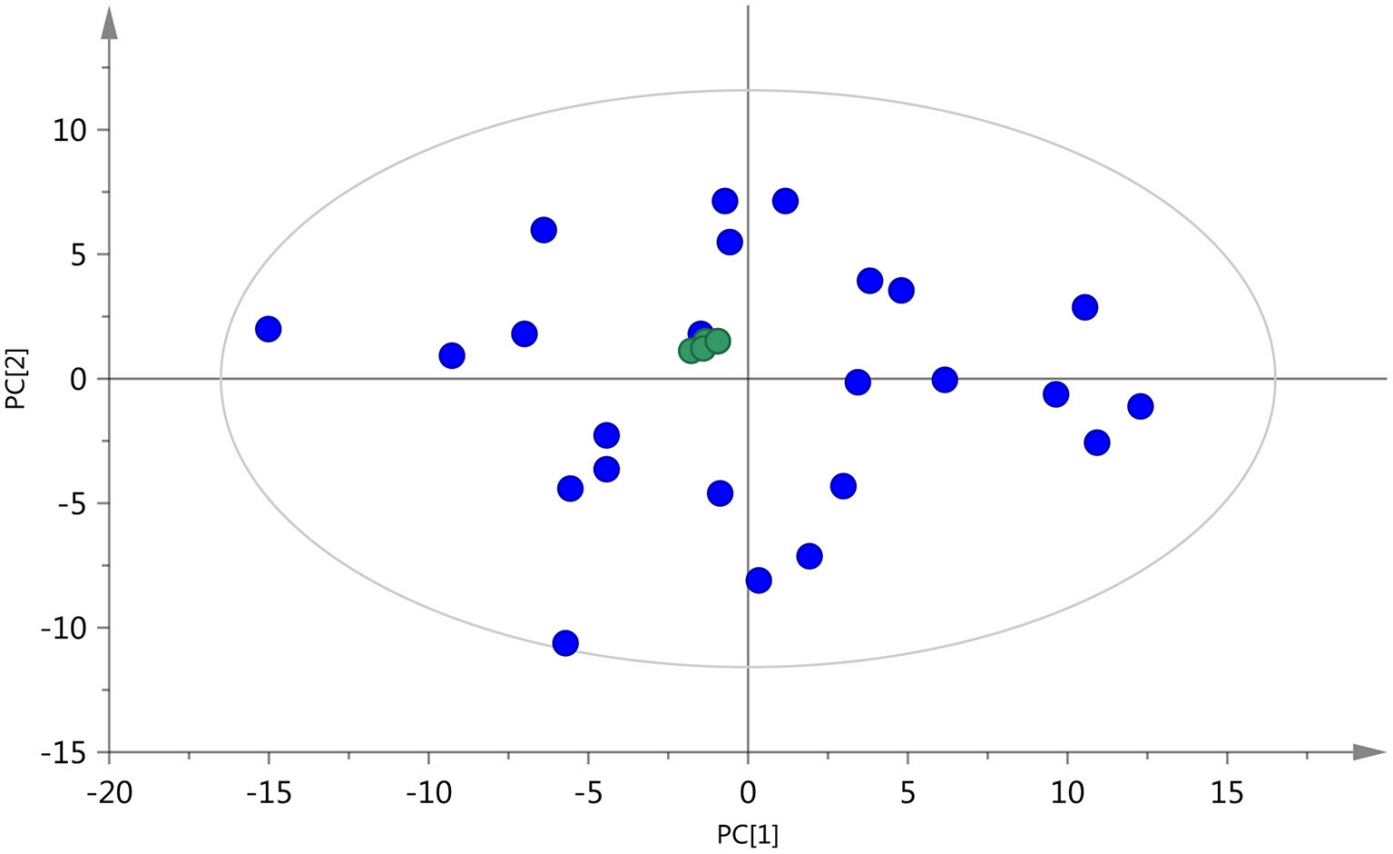
PCA score plot of test samples and quality control (QC) samples (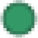, QC sample; 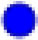 test sample).

The accuracy and reproducibility of the analysis process were monitored using QC samples, which were inserted for every five test samples, to obtain high-quality mass spectrum data. The retention time and the peak area of QC samples in the total ion current graph overlapped well, indicating that the instrument was stable (Supplementary Figure S1). As shown in Figure 2, the distribution of QC samples was dense and close to the center in the PCA score plot, and the QC samples were all within two standard deviations (Supplementary Figure S2), indicating good stability of the method and high quality of the data (28). Moreover, all the samples were within a 95% confidence interval, which also showed that the experimental data had no outlier in the dataset.

### Qualitative and Quantitative Metabolites

In the present study, 174 peaks were detected and 159 peaks were left after using the relative standard deviation de-noising and total ion current normalization method. The qualitative and quantitative analyses of metabolites in samples were performed based on the in-house MS2 database, KEGG database, HMDB database, and MRM. A total of 159 metabolites were detected and quantified with the widely targeted metabolite technique, which included 26 carbonyls, 20 organic acids and its derivatives, 18 amino acid and its derivatives, 14 glucosides, 12 alcohols and polyols, 11 flavonoids, 5 nucleotide and its derivatives, 5 glycerophospholipids and fatty acyls, three vitamins, 13 benzene and substituted derivatives, and 32 other metabolites including catechol derivatives, choline and derivatives, pyridine derivatives, tetrapyrroles derivatives, and others. The information and content of the identified metabolites is shown in Supplementary Table S1.

### Discrimination of Peach Origins

**Figure 3:**
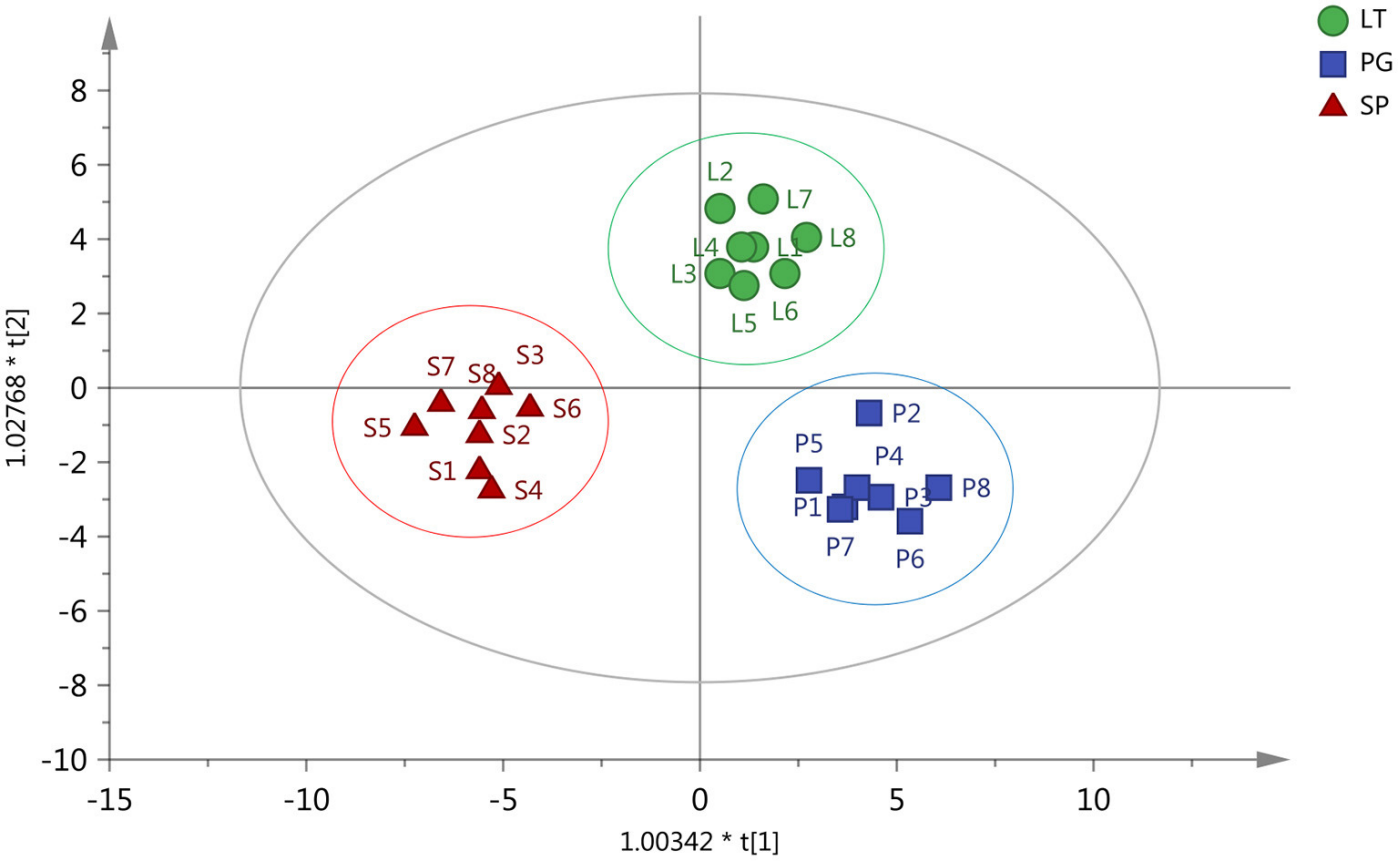
Orthogonal partial least squares-discriminant analysis (OPLS-DA) test for the identification of the three regions of peach samples. PG (Pinggu, Beijing 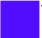), SP (Shunping, Hebei 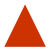), and LT (Laoting, Hebei 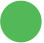).

**Figure 4:**
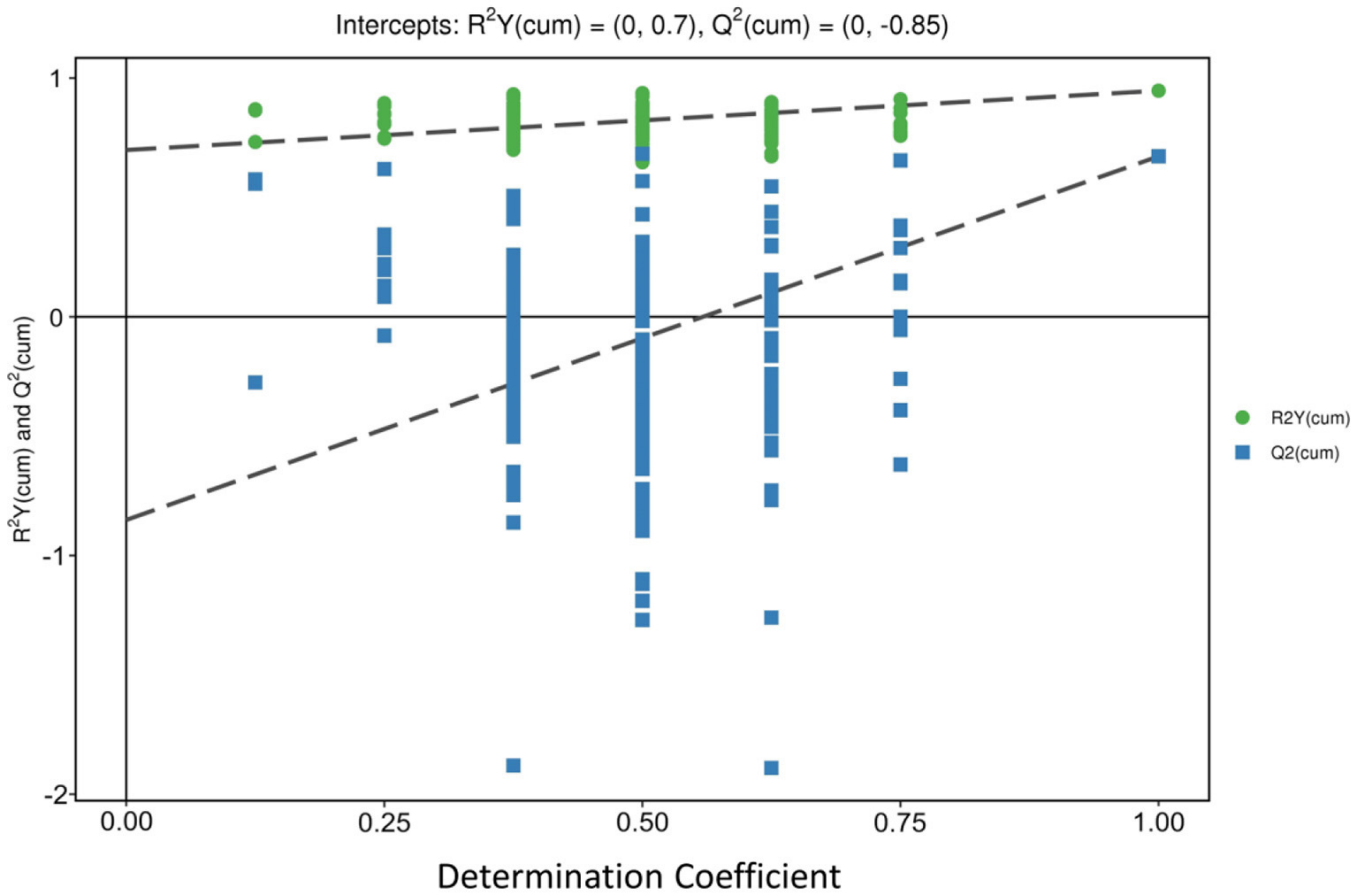
Permutation test of OPLS-DA model for Pinggu group vs. Shunping group.

The orthogonal partial least squares-discriminant analysis (OPLS-DA) decomposes the X matrix information into Y correlation and irrelevance by orthogonal signal correction and partial least squares-discriminant analysis (PLS-DA), which can filter out the variables not related to classification and can maximize group differentiation and help find differential metabolites (36). OPLS-DA was performed to determine whether the three regions of peach samples could be differentiated. The results of the OPLS-DA showed three distinctive groups: LT group, PG group, and SP group. All samples were within 95% Hotelling's T-squared ellipse (Figure 3). The three groups were clearly separated, indicating differences in metabolic profiles. The permutation test was performed using 200 alignment experiments to evaluate the statistical significance of the OPLS-DA model. The permutation test result using an example of group PG vs. SP is shown in Figure 4. The green and blue dots represented the R^2^Y or Q^2^ after replacement, and the two dotted lines represented the regression lines of R^2^Y or Q^2^. The value of R^2^Y was very close to one, and the value of Q^2^ was closer to one, which showed the established modules corresponding to the real situation of the samples. A relatively approximate distribution would be obtained if a new sample was added to the model. The result confirmed that the model was meaningful, and the original model could well explain the differences between the two groups of samples.

The number of the variable important in the projection (VIP value) greater than one was up to 62 with further analysis of the metabolic profiles. The information of the 62 compounds is shown in Supplementary Table S2. The largest number of organic acids and its derivatives was 11, including quinic acid, malic acid, and so forth. The next largest number of carbonyls was 10, followed by prenol lipids and benzene and substituted derivatives in the third place (the number was seven). The number of carboxylic acids and derivatives was five. A VIP value higher than one indicated an important variable to the OPLS-DA model. If the same explanations existed between independent variables and y, the VIP values would be one. The higher the value, the greater the independent variable's contribution to the model (37). Therefore, the relatively large number of VIP>1 (about 39%) also reflects the large similarity among metabolites in peach within a small geographical range, which, to some extent, explains why it is difficult to differentiate the agricultural products from small-scale origins, so that many current analytical methods cannot accurately distinguish them.

### Screening of Differential Metabolites

**Figure 5:**
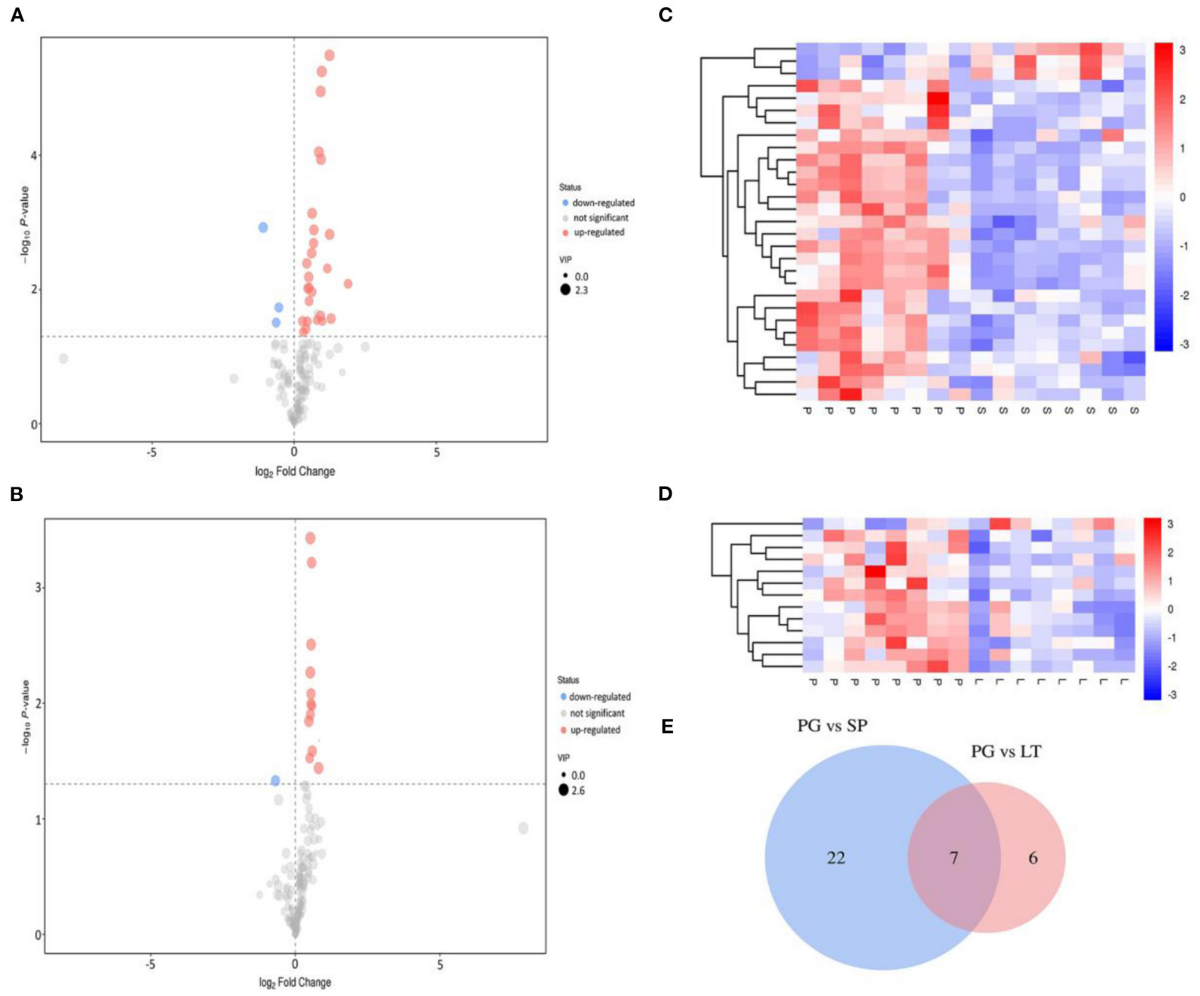
Volcano plot and hierarchical cluster analysis (HCA), and Venn diagram. **(A,B)** represent the volcano plot showing the differential expression of metabolites between PG and SP, and PG and LT, respectively. The red dots in the figure represent the differentially expressed metabolites that were increased, the blue dots represent the decreased differentially expressed metabolites, and the gray color indicates the differentially expressed metabolites that were not significant. **(C,D)** represent the heat map showing the differential expression of metabolites between PG and SP, and PG and LT, respectively. The red color indicates the increase in differentially expressed metabolites, while the blue color indicates the decrease in differentially expressed metabolites. **(E)** is the Venn diagram showing the specific and common differential metabolites between PG and SP, and PG and LT.

Compared with the traditional univariate analysis (UVA), such as the Student *t-*test or analysis of variance, the multivariate data analysis pays more attention to the correlations between several variables simultaneously and analyzes the statistical rules of data sets under multiple objects and variables related to each other (38). Student *t*-test and OPLS-DA were simultaneously applied to find the differential metabolites between different groups. The metabolites with a *P-*value <0.05 and a VIP >1 were selected and regarded as significant differential metabolites. The volcano plot, HCA and Venn diagram were employed to further analyze and clearly show the characteristics of the significant differential metabolites from different regions of peach samples. The results are shown in Figure 5. The Venn diagram (Figure 5E) showed 29 (PG vs SP) and 13 (PG vs LT) kinds of significantly different metabolites, of which seven were common. As shown in Figures 5A,B, most of the metabolites had no significant changes in the pairwise comparisons; for PG vs. SP, 26 metabolites were upregulated while three metabolites were downregulated; for PG vs. LT, 12 metabolites were upregulated while one metabolite was downregulated. As shown by HCA results in Figures 5C,D, on the whole, different metabolites were obvious between groups, and the variation pattern within groups was basically the same. Thus, the result further confirmed that the metabolic profiles were feasible to distinguish peach from small-scale districts.

### KEGG Annotation and Enrichment Analysis of Differential Metabolites

**Figure 6:**
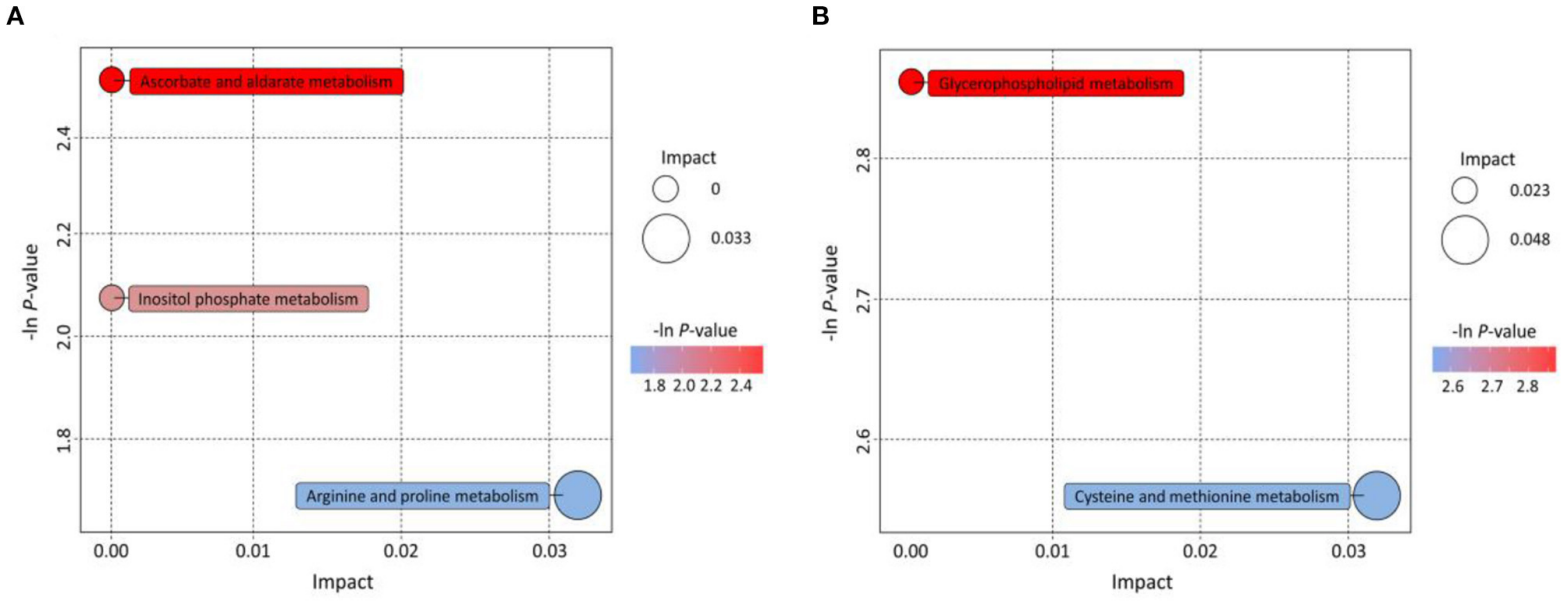
KEGG annotations and enrichment of differentially expressed metabolites of each pairwise comparison of peach. **(A)** PG vs. SP; **(B)** PG vs. LT. Bubbles represent metabolic pathways, and the size and horizontal coordinate of the bubble represent the influence factor in the pathway. The larger the bubble size, the greater the impact factor. The longitudinal coordinate and the color of the bubble represent the *P*-value of enrichment analysis. The deeper the color and the smaller the *P*-value represent more significant enrichment degree.

The KEGG pathway database is the most commonly used database in the metabolic pathway and regulatory pathway studies in general. The analysis of metabolic pathways helps systematically understand the impact of the habitat environment on bionts, and provides some good references for further exploring the adaptation of plants to the environment (39). The pathways of differential metabolites mapping to KEGG were determined through pairwise contrasts of significant differential metabolites among regions. Based on the metabolites annotated to the metabolic pathways, 14 (48.28%) and 6 (46.15%) metabolites, respectively, coming from PG vs. SP and PG vs. LT were successfully annotated to the KEGG pathways. The results of the KEGG enrichment analysis are shown in Figure 6. The bubble plots displayed the enriched metabolic pathway, while each bubble represented a metabolic pathway. Figure 6A shows three significantly enriched pathways compared with PG vs. SP: ascorbate and aldarate metabolism, inositol phosphate metabolism, and arginine and proline metabolism. The arginine and proline metabolism pathways possessed a greater impact factor. The two enriched metabolic pathways were glycerophospholipid metabolism and cysteine and methionine metabolism compared with PG vs. SP. As shown in Figure 6B, cysteine and methionine metabolism pathways had a greater impact factor. Hence, differences in amino acid metabolism were found between the samples from Pinggu District and those from the other two habitats. Not only are amino acids the main components of proteins, but also the variety and contents of amino acids are important parameters to their nutritional quality and sensory taste in fruits (40). Besides, amino acids participate in many metabolic networks that control growth and adaptation to the environment; some amino acid metabolism pathways are the signals of plant response to the environment (41). Furthermore, evidence showed that arginine could promote the biosynthesis of proline, polyamines, and nitric oxide to improve cold tolerance in plants (42). Meanwhile, a previous study showed that proline possessed many biological functions, such as maintaining the osmotic balance, stabilizing the protein structure, clearing of oxygen-derived free radicals, and restraining membrane lipid peroxidation; it also had certain effects in postharvest fruits to cope with chilling injury (43). Liao et al. assessed the roles of cysteine and methionine in the response of poplar leaf to salt stress. They found that the salt-triggered H_2_S-cysteine cycle and methionine-associated ethylene synthesis induce the alternative oxidase pathway to scavenge reactive oxygen species by cooperating with antioxidant enzyme systems (44). Therefore, different habitats were associated with different agricultural product quality, which is the characteristic of GI products. The results also showed that our widely targeted metabolomics method was effective in discriminating peaches from different regions.

### Evaluation of Method Greenness

**Figure 7:**
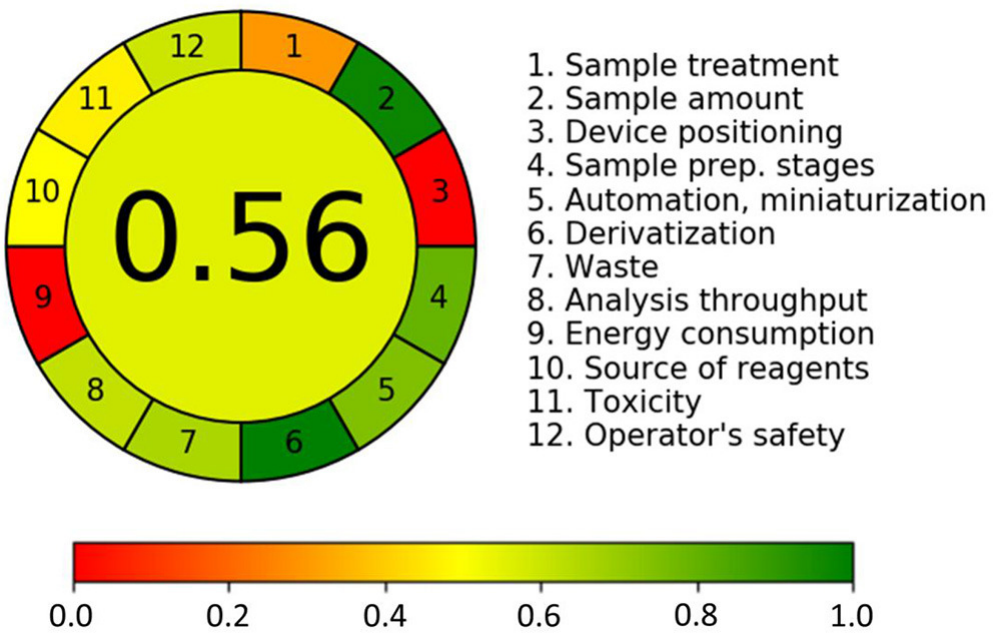
Greenness of the developed method was assessed with Analytical GREEnness metric approach (AGREE); annotated result of a generic assessment (above) and the corresponding color scale for reference (below).

The greenness of the analytical methods was assessed using green analytical chemistry (GAC). GAC is the concept that makes analytical chemists consider the environmental, health, and safety issues during their activities (45). The greenness of methods is gradually being taken seriously by researchers. The Analytical GREEnness metric approach (AGREE) is a novel, easily interpretable, and informative assessment approach (46). In our study AGREE tools were applied to evaluate the greenness of the developed analytical method. The output of the GREEnness metric approach is a clock-like graph, with the overall score and color representation in the middle. The final assessment, as in Figure 7, showed the overall light green–colored pictogram with a numerical value of 0.56, which illustrated that the widely targeted metabolomics method was relatively green. In the 12 AGREE segment, the value closer to one and dark green color indicated that the assessed procedure was green, while the value of zero and yellow color indicated that the corresponding procedure needed improvement and amelioration. For the Analytical GREEnness report sheet for this study associated with Supporting Information, please refer to Appendix 2.

## Discussion

Food authenticity, including geographical origin, planting pattern (conventional and organic), or animal origin, has become increasingly important, directly influencing people's consumption willingness and happiness indices. In particular, people have higher psychological expectations for high-end and regional characteristic products, which have a higher price and often more serious counterfeiting. Ensuring food authenticity is an important challenge faced by researchers, regulatory bodies, and industries. The accuracy of geographical origin is an important part of food authenticity, which has always been a hot research issue. At present, many researches are devoted to this area, and the related technologies are also diverse. However, great challenges in distinguishing agricultural products from adjacent origins remain. Researchers combined multiple techniques, such as stable isotopes, elements, and amino acids, to trace the origin of milk from different districts in Inner Mongolia (47). However, the combination application of technologies not only requires more experimental equipment and complicated experimental operation but also increases error factors and test costs. Besides, the simultaneous application of more technologies certainly increases the difficulty in popularization and application to a certain extent. Furthermore, the distinction effect is still not very ideal for the regional products from small-scale districts.

In our study, peach samples were collected from Beijing Pinggu District, which is the major peach-producing area. The other two sampling sites were located in Hebei province, which are adjacent peach-producing areas to Pinggu District. The straight line distance among Pinggu District and the other two sampling sites was about 200 km. All the three sites belonged to the same cumulative temperature zone, but with some climatic differences. The UHPLC-MS/MS was applied to determine the geographical origin of peach samples from three adjacent origins. This technology had not only super-sensitivity but also adequate repeatability, and the experimental results were stable and reliable. Based on differential metabolite screening in combination with multivariate statistical analysis, this method achieved good performance in differentiating the peach samples from small-scale districts. Also, the prediction model was stable and reliable, and could be used to classify the origins of samples based on identified patterns.

However, this study also had some aspects for further in-depth research and improvement. First, the differences in the metabolite expression profiles of peach samples from different geographical origins were confirmed by this study, but the reasons for the differences are still ambiguous. Besides, the agronomic measures or production management also affects the physiological metabolism of plants to some extent. Therefore, further studies specifically aimed at the effect of the environmental factors on the metabolic expression profile alone should be performed for ensuring the consistency of agronomic measures to further clarify the reasons for the differences in metabolite expression profiles. In addition, the high-resolution mass spectrometry was first used to establish the MRM mass spectrometry library, and then the high-sensitivity mass spectrometry was applied to quantify metabolites in this study. This technological process helped improve the accuracy of metabolite characterization and quantification. It also narrowed the number of metabolites obtained and lost some metabolite information to a certain extent, leaving some room for subsequent improvement to this method. Anyway, the widely targeted metabolomics results showed that the kind and number of identified metabolites were also satisfactory for the differentiation of samples from different origins.

At present, metabolomic techniques based on different principles have been increasingly reported in the field of product origin traceability and authenticity identification. The unique advantages of this technology definitely provide more room for development in the future.

## Conclusions

The results of this study confirmed that the metabolites based on widely targeted metabolomics could be used as fingerprints to discriminate the geographical origin of peach samples from small-scale districts. In addition, the representative metabolites with VIP value >1 made larger contributions to the distinguishing model with OPLS-DA. In conclusion, the proposed method was a more effective method to combine the widely targeted metabolomics and OPLS-DA for the identification of the geographic origins of peach samples from small-scale origins. The method could help further protect the geographical indications, protected designation of origin, and the regional characteristic products.

## Data Availability Statement

The datasets presented in this study can be found in online repositories. The names of the repository/repositories can be found in the article/Supplementary Material.

## Author Contributions

JZ: investigation, methodology, formal analysis, visualization, and writing. AL, XJ, and GL: resources. LP: supervision and project administration. All authors contributed to the article and approved the submitted version.

## Funding

The National Natural Science Foundation of China (32102057) and the Beijing Natural Science Foundation (6194038) supported to the samples collection and metabonomics analysis. Besides, the China's Post-doctoral Science Fund (2019M650554) also provided fund support. The publication fees will be provided by our institution.

## Conflict of Interest

The authors declare that the research was conducted in the absence of any commercial or financial relationships that could be construed as a potential conflict of interest.

## Publisher's Note

All claims expressed in this article are solely those of the authors and do not necessarily represent those of their affiliated organizations, or those of the publisher, the editors and the reviewers. Any product that may be evaluated in this article, or claim that may be made by its manufacturer, is not guaranteed or endorsed by the publisher.
