# Supplementary material for: Discrimination of Geographical Origin of Agricultural Products From Small-Scale Districts by Widely Targeted Metabolomics With a Case Study on Pinggu Peach
Source: Front Nutr. 2022 May 24;9:891302. doi: 10.3389/fnut.2022.891302 (PMC9172448; doi:10.3389/fnut.2022.891302)
Supplement: Supplementary file 1 [file Data_Sheet_1.pdf]

Supplementary material

Discrimination of geographical origin of agricultural products from small-scale districts by widely targeted metabolomics with a case study on Pinggu Peach

Jie Zhao[a,b], An Li[a,b], Xinxin Jin[a,b], Gang Liang[a,b], Ligang Pan[a,b]*

[a] Institute of Quality Standard and Testing Technology, Beijing Academy of Agriculture and Forestry Sciences, Beijing 100097, China

[b] Ministry of Agriculture, Risk Assessment Lab for Agro-products (Beijing), Beijing 100097, China

*Corresponding author: panligang2012@126.com

Page Information

Figure S1. Total ion current (TIC) diagram of positive and negative mode for QC samples

Figure S2. One dimensional distribution diagram of PCA-X for quality control (QC) samples in PCA scores plot.

Table S1. The information and content of the identified metabolites.

Table S2. Metabolites with the value of the most important discriminant variables greater than one.

Figure S1

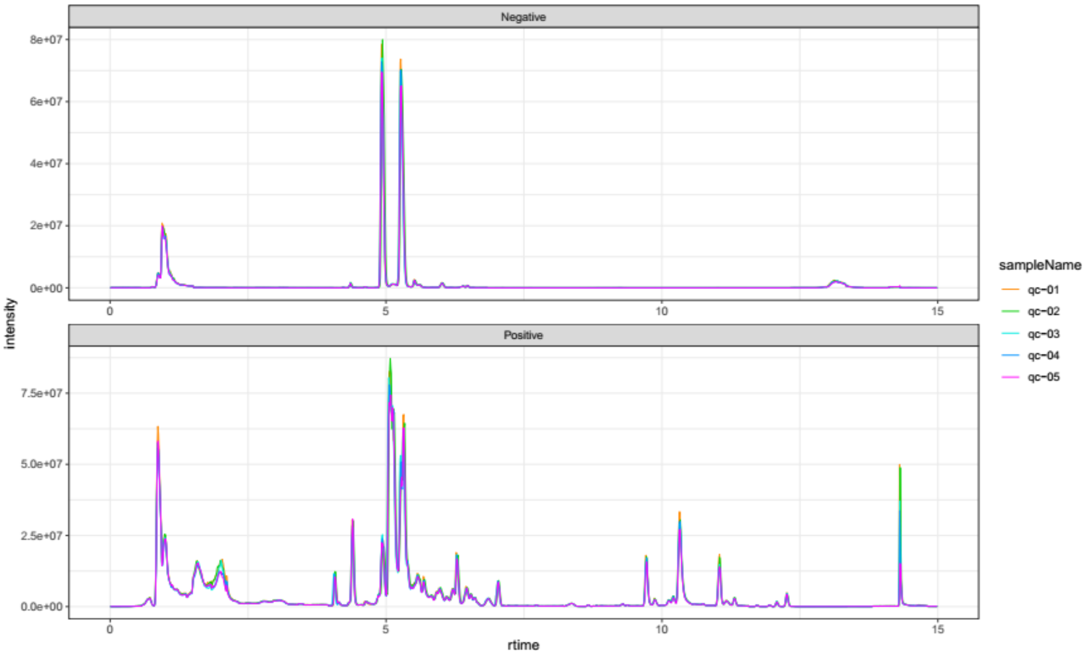

Negative
8e+07
6e+07
4e+07
2e+07
0e+00
0
5
10
15
intensity
Positive
7.5e+07
5.0e+07
2.5e+07
0.0e+00
rtime
sampleName
qc-01
qc-02
qc-03
qc-04
qc-05

Figure S2

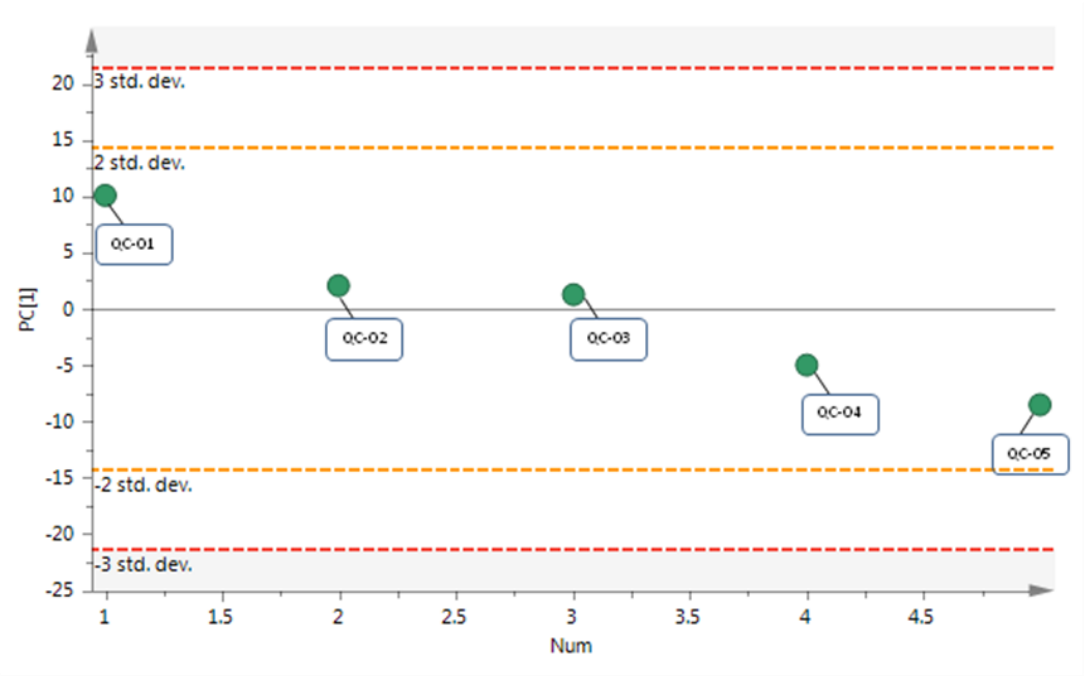
3 std. dev.
2 std. dev.
-2 std. dev.
-3 std. dev.
QC-01
QC-02
QC-03
QC-04
QC-05
PC[1]
Num
20
15
10
5
0
-5
-10
-15
-20
-25
1
1.5
2
2.5
3
3.5
4
4.5

Table S1

No.	compound name	Class	polarity	RT	Q1	Q3	Group QC	Group L	Group P	Group S
1	β-Terpinyl acetate		+	6.64	197.153	179.142	4.34737E-05	5.16084 E-05	5.67105 E-05	4.24785 E-05
2	α-Linolenoyl Ethanolamide		+	10.75	322.274	62.06	0.000957621	0.00060 301	0.00093 2501	0.00051 4886
3	Valsartan acid		+	4.16	267.096	150.055	0.001474399	0.00195 0551	0.00210 6271	0.00161 2914
4	Valine		+	1.43	118.086	55.054	0.002002786	0.00207 8132	0.00195 25	0.00172 5916
5	Triphenylphosphate		+	10.32	327.078	152.061	0.000776127	0.00072 6707	0.00108 866	0.00082 7242
6	Tri(butoxyethyl)phosphate		+	10.8	399.25	299.16	1.53922E-05	1.52482 E-05	1.79701 E-05	1.69431 E-05
7	Thymoquinone	Organooxygen compounds	+	6.13	165.091	137.096	0.000429689	0.00045 7304	0.00050 9068	0.00036 4976
8	Threonine		+	0.87	120.065	74.06	9.80306E-05	0.00010 7979	0.000113 29	8.77321 E-05
9	Threonic acid	Organooxygen compounds	-	0.91	135.03	75.009	0.000603775	0.00055 447	0.00072 3086	0.00073 7754
10	Tetradecanedioic acid	Fatty Acyls	+	5.94	259.189	67.054	9.53935E-05	8.86047 E-05	0.00010 322	9.59395 E-05
11	sweroside		+	4.83	359.131	197.078	0.000433305	0.00050 286	0.00053 4551	0.00022 5868
12	Sucrose	Organooxygen compounds	+	14.32	365.106	203.052	0.035038839	0.03335 188	0.03042 3776	0.02658 53
13	Salicylic acid	Benzene and substituted derivatives	-	5.18	137.024	93.034	0.001103808	0.00138 7143	0.00171 0417	0.00086 4384
14	S-Adenosylmethionine	5'-deoxyribonucleosides	+	0.93	399.144	250.093	0.010629741	0.00918	0.011263	0.01051

								6436	636	9917
15	Quinic acid	Organooxygen compounds	-	0.98	191.056	85.029	0.081337206	0.08043 2334	0.08296 1568	0.09289 2458
16	Quercetin 3-galactoside	Flavonoids	-	6.03	463.088	300.027	0.002352049	0.00240 0952	0.00223 8705	0.00209 5655
17	Pyrrolidonecarboxylic acid	Carboxylic acids and derivatives	+	0.88	130.05	84.044	0.009439381	0.00864 3927	0.00830 2429	0.01206 8259
18	p-Xylene	Benzene and substituted derivatives	+	5.62	107.085	91.054	0.000833771	0.00087 7939	0.00095 7072	0.00061 8668
19	Pseudoionone	Prenol lipids	+	8.36	193.158	175.148	8.85341E-05	9.43441 E-05	0.00010 3038	9.48766 E-05
20	Procyanidin C1	Flavonoids	-	4.43	865.199	125.024	0.000208723	0.00016 2087	0.00016 7463	0.00014 2633
21	Procyanidin B2	Flavonoids	+	5.7	579.15	127.038	0.005029342	0.00558 6479	0.00457 3623	0.00475 806
22	Procyanidin B1	Flavonoids	+	4.44	579.15	127.038	0.000782026	0.00078 4898	0.00065 6088	0.00056 1201
23	p-Mentha-1,3,8-triene	Prenol lipids	+	5.62	135.116	107.085	0.000120418	0.00013 9887	0.00013 308	0.000114 148
24	p-Mentha-1,3,5,8-tetraene	Benzene and substituted derivatives	+	6.42	133.101	105.07	0.001389542	0.00092 8954	0.00162 3629	0.00085 9405
25	Phytosphingosine	Organonitrogen compounds	+	8.82	318.3	60.044	0.000617724	0.00046 852	0.00064 6051	0.00071 3154
26	Phosphoenolpyruvic acid	Organic phosphoric acids and derivatives	-	0.77	166.975	78.959	4.41687E-05	6.15493 E-05	5.4232E- 05	4.37638 E-05
27	Phlorizin	Flavonoids	-	5.82	435.13	273.077	7.21574E-05	6.42109 E-05	6.43134 E-05	0.000116 495
28	Perillyl acetate	Prenol lipids	+	5.96	195.137	93.07	0.000660089	0.00099 743	0.00156 3983	0.00063 5512
29	PC(16:0/0:0)[U] / PC(16:0/0:0)[rac]		+	10.32	496.34	184.073	0.079294351	0.06795	0.07930	0.114497

								3328	164	061
30	PAz-PC		+	11.17	666.433	184.073	0.00458365	0.00616 126	0.00733 8059	0.00497 5656
31	Pantothenic acid	Alcohols and polyols	+	4.62	220.117	90.055	0.001992895	0.00220 4535	0.00257 6972	0.00203 05
32	Palmitic amide	Fatty Acyls	+	12.09	256.263	88.075	0.003189555	0.00155 5583	0.00202 6223	0.00174 7968
33	Oleamide	Fatty Acyls	+	12.27	282.278	69.07	0.008842254	0.00410 585	0.00391 8193	0.00398 2895
34	o-Ethyltoluene	Benzene and substituted derivatives	+	6.21	121.101	93.07	0.000169796	0.00015 3444	0.00021 9999	0.00012 039
35	Octopine		+	7.11	247.132	229.121	5.11078E-05	0.00010 0965	6.70014 E-05	5.97628 E-05
36	N-Oleoylethanolamine	Organonitrogen compounds	+	11.95	326.304	62.06	0.000896733	0.00085 8491	0.00158 7502	0.00042 6302
37	Niacinamide	Pyridines and derivatives	+	2.05	123.055	80.049	0.000786046	0.00076 0513	0.001140 34	0.00104 3342
38	NCGC00381407-01		+	5.75	265.142	247.132	2.79039E-05	2.62792 E-05	3.52197 E-05	3.16103 E-05
39	N-Benzylformamide		+	3.09	136.075	91.054	0.002700925	0.00289 121	0.00255 7457	0.00297 9522
40	N6-Acetyl-L-lysine	Carboxylic acids and derivatives	+	1.63	189.123	84.08	0.002740448	0.00293 2745	0.00235 5088	0.00247 3643
41	Miscanthoside	Flavonoids	-	6.04	449.109	287.056	0.000288896	0.00030 9936	0.00019 3064	0.00020 2707
42	Methylsyringin	Organooxygen compounds	+	5.82	387.164	225.108	0.000347823	0.00031 5278	0.00028 2384	0.00026 8717
43	Methyl cinnamate	Cinnamic acids and derivatives	+	5.52	163.075	131.049	0.000245007	0.00024 0998	0.00032 9722	0.00029 4337
44	Melezitose	Organooxygen compounds	-	0.99	503.161	89.024	0.001292584	0.00145	0.00152	0.00124

								9819	7452	0606
45	Mannitol	Organooxygen compounds	-	0.89	181.072	71.014	0.008664331	0.00966 7924	0.01005 9749	0.00851 7136
46	Malic acid	Hydroxy acids and derivatives	-	1.06	133.014	115.004	0.000135073	0.00015 1549	0.00017 2165	0.00012 4117
47	LysoPE(18:1(9Z)/0:0)	Glycerophospholipids	+	10.3	480.308	339.288	0.003826832	0.00387 9316	0.00582 7104	0.00517 7733
48	LysoPE(16:0/0:0)	Glycerophospholipids	+	10.21	454.293	313.272	0.00625669	0.00482 4661	0.00692 6246	0.00881 5635
49	L-Tyrosine	Carboxylic acids and derivatives	+	3.09	182.081	136.075	0.000313606	0.00036 0033	0.00032 448	0.00038 5688
50	Linoleoyl ethanolamide	Organonitrogen compounds	+	11.32	324.289	62.06	0.005006825	0.00478 5914	0.00516 6536	0.00354 1046
51	Licoagroside B	Saccharolipids	+	1	433.133	127.039	0.00087234	0.00091 271	0.00095 5326	0.00096 9694
52	L-Histidine	Carboxylic acids and derivatives	+	0.86	156.076	110.071	0.00076305	0.00097 4493	0.00084 2273	0.00042 9232
53	L-beta-Homothreonine		+	0.88	134.081	74.023	0.000858733	0.00100 3994	0.00096 617	0.00077 469
54	Isoquercitrin	Flavonoids	+	5.91	465.103	303.049	0.00044764	0.001181 639	0.00126 826	0.00137 4433
55	Isocitric acid	Carboxylic acids and derivatives	-	1.04	173.009	111.009	0.000182966	0.00021 0382	0.00030 4743	0.00018 7338
56	Iridotrial		+	6.13	183.101	137.096	0.000498487	0.00053 0122	0.00058 8049	0.00041 0094
57	Hymecromone methyl ether		+	5.76	191.07	91.054	0.001015299	0.00107 8725	0.00122 5801	0.00099 8983
58	Histidine		-	0.83	154.062	93.046	4.68573E-05	5.57946 E-05	3.6297E-05	2.92138 E-05
59	Glycerophosphocholine	Glycerophospholipids	+	0.88	258.109	104.107	0.040262997	0.07402	0.04597	0.02866

								954	3063	4502
60	Glucose 1-phosphate	Organooxygen compounds	-	0.88	259.022	78.959	0.001979457	0.00274 8325	0.00212 294	0.00240 687
61	Glucitol-4-gucopyanoside		-	0.97	343.124	59.014	0.003319475	0.00337 7775	0.00393 0083	0.00342 3322
62	Gentisate aldehyde	Organooxygen compounds	+	0.98	139.039	65.039	0.001229787	0.00129 7059	0.00129 8083	0.00133 8603
63	Galactaric acid	Organooxygen compounds	-	0.89	209.03	85.029	0.000294224	0.00042 0365	0.00036 9585	0.00025 7299
64	Gabapentin	Carboxylic acids and derivatives	+	11.36	172.133	109.101	2.07327E-05	7.02838 E-06	6.66894 E-06	1.11456 E-05
65	Ethiin	Carboxylic acids and derivatives	+	4.39	166.049	120.08	0.026620947	0.02707 5596	0.02607 4708	0.03031 0778
66	eriodictyol-7-O-glucoside		-	6.41	449.109	151.004	0.000591653	0.00057 136	0.00059 1453	0.00059 2059
67	Egomaketone	Carbonyl compounds	+	5.61	165.091	95.012	0.000582642	0.00060 0202	0.00064 7348	0.00045 4363
68	Docosahexaenoic Acid ethyl ester		+	9.88	357.278	105.069	1.41436E-05	9.82848 E-06	1.50161 E-05	6.29502 E-06
69	DL-Glutamate	Carboxylic acids and derivatives	+	0.89	148.06	84.044	0.020854082	0.01915 3708	0.02039 1217	0.02614 4157
70	Dimethomorph		+	8.76	388.131	301.061	0.000314122	3.63451 E-06	1.96611 E-06	0.00054 634
71	Dibutyl phthalate	Benzene and substituted derivatives	+	11.04	279.158	149.023	0.029320296	0.03281 9701	0.03382 7956	0.02953 7127
72	D-Glutamine	Carboxylic acids and derivatives	+	0.87	147.076	84.044	0.01089192	0.00940 6808	0.00849 9481	0.01321 5974
73	D-Glucuronic acid	Organooxygen compounds	-	0.88	193.035	103.004	5.79512E-05	6.38955 E-05	6.72275 E-05	5.49151 E-05
74	Dehydrovomifoliol	Prenol lipids	+	6.57	223.132	205.122	0.000562982	0.00069	0.00066	0.00060

								1542	3266	2518
75	Cytosine	Diazines	+	1.74	112.05	52.018	0.000426521	0.000384005	0.000632147	0.000789783
76	Cytidine	Pyrimidine nucleosides	+	1.75	244.092	112.05	0.002429844	0.002022616	0.003473687	0.004512872
77	Cyanidin 3-glucoside	Flavonoids	+	5.14	449.107	287.054	0.224646268	0.193354354	0.181286097	0.139862651
78	Citrulline	Carboxylic acids and derivatives	+	0.9	176.102	70.065	0.002359606	0.001518893	0.002124311	0.004534589
79	Citral propylene glycol acetal	Dioxolanes	+	8.36	211.169	67.054	0.004946795	0.005304384	0.005865843	0.005416206
80	Cis-Zeatin-9-glucoside		+	4.62	382.17	220.117	6.03385E-05	7.01261E-05	8.09652E-05	4.72079E-05
81	Choline	Organonitrogen compounds	+	0.86	104.107	60.081	0.022333406	0.02355491	0.025418221	0.023497612
82	cholesta-3,5-dien-7-one		+	13.25	383.33	175.148	2.66195E-06	1.91526E-06	3.09791E-06	2.63972E-06
83	Chlorogenic acid	Organooxygen compounds	+	4.33	355.099	163.038	0.000360528	0.000337617	0.00048717	0.000404405
84	Catechin	Flavonoids	+	5.34	291.086	139.039	0.098110078	0.106402708	0.103218368	0.105098201
85	Caffeic acid	Cinnamic acids and derivatives	+	5.3	181.049	89.038	0.000816581	0.000996732	0.000955086	0.000962088
86	Biliverdin	Tetrapyrroles and derivatives	+	8.66	583.255	297.123	0.001045854	0.000942647	0.001155537	0.000664708
87	Betaine	Carboxylic acids and derivatives	+	0.9	118.086	58.065	0.009154263	0.011291268	0.011933917	0.010790474
88	beta-D-Glucopyranosyl anthranilate	Organooxygen compounds	+	4.67	300.108	138.055	4.52945E-05	0.000116532	4.94604E-05	6.6222E-05
89	beta-D-Glucopyranoside, 4-(3-hydroxypropyl)-2-methoxyphenyl		+	5.21	362.18	183.101	3.62143E-05	3.95132	4.77492	3.6237E-

								E-05	E-05	05
90	beta-D-Glucopyranoside, 2-phenylethyl 2-O-beta-D-xylopyranosyl-		+	5.85	434.201	85.028	0.001538522	0.001839849	0.00209037	0.001829632
91	beta-D-Glucopyranose, 1-O-[(2E,6E)-8-hydroxy-2,6-dimethyl-1-oxo-2,6-octadien-1-yl]-		+	6.45	369.152	207.099	0.001208875	0.002038448	0.001364258	0.001064275
92	beta-D-Glucopyranose, 1-O-[(2E)-3-(2-hydroxyphenyl)-1-oxo-2-propen-1-yl]-		+	6.46	309.097	147.044	0.003056341	0.003388106	0.002719008	0.004323744
93	Benzyl 6-O-beta-D-glucopyranosyl-beta-D-glucopyranoside		+	5.23	450.196	91.054	0.000548511	0.00076134	0.000576328	0.000178166
94	Benzyl 6-O-(6-deoxy-alpha-L-mannopyranosyl)-beta-D-glucopyranoside		-	5.83	461.167	415.161	9.52509E-06	1.45547E-05	1.37819E-05	1.15812E-05
95	Naphtho[2,3-b]furan-9(4H)-one, 4,8-bis(acetyloxy)-4a,5,6,7,8,8a-hexahydro-3,4a,5-trimethyl-, (4S,4aR,5S,8S,8aS)-		+	5.98	331.154	151.075	0.002064433	0.002312166	0.002888751	0.002375504
96	Azoxystrobin		+	9.3	404.124	372.096	0.001209123	5.69595E-06	0.001353394	0.005897379
97	Astragalin	Flavonoids	-	5.13	447.093	284.032	0.002578988	0.003062787	0.002504187	0.001438734
98	Ascorbic acid	Dihydrofurans	-	1.05	175.025	87.009	0.000142734	0.000153209	0.00028897	9.92346E-05
99	Amygdalin	Organooxygen compounds	-	5.41	456.151	323.098	1.24527E-05	5.47186E-06	7.52831E-06	7.40401E-06
100	alpha-D-Glucopyranoside, alpha-D-glucopyranosyl, 2-(2-methylbutanoate)		+	14.37	449.155	287.054	3.49678E-05	3.68617E-05	3.73306E-05	2.75358E-05
101	Allysine	Carboxylic acids and derivatives	+	0.9	146.081	100.047	0.002253161	0.001167499	0.001215703	0.001642716
102	Allantoic acid	Carboxylic acids and derivatives	+	5.41	177.054	89.038	0.004024751	0.004207539	0.005302512	0.004837821
103	Adenosine_Diphosphate		+	1.36	428.035	136.061	0.000750181	0.000936363	0.001119819	0.001180007

104	Adenosine	Purine nucleosides	+	4.06	268.103	136.061	0.018134488	0.020528208	0.019309512	0.028731388
105	Adenine	Imidazopyrimidines	+	1.64	136.061	119.035	0.015149662	0.01289498	0.014866004	0.016563971
106	9,11-methane-epoxy PGF1α		+	11.21	353.268	261.22	0.000453602	0.000462339	0.000461015	0.000485806
107	6,10-Dimethyl-5(E),9-undecadien-2-one		+	6.2	195.174	177.163	0.0001141	9.97694E-05	0.000145959	7.65227E-05
108	5'-Methylthioadenosine	5'-deoxyribonucleosides	+	4.96	298.096	136.061	0.01443909	0.013451473	0.019582059	0.016809284
109	5-Caffeoylquinic acid		+	5.29	355.102	163.038	2.40584E-07	3.64527E-07	3.07004E-07	5.42069E-08
110	5-[6-(3-hydroxy-4-methoxyphenyl)-1,3,3a,4,6,6a-hexahydrofuro[3,4-c]furan-3-yl]-2-methoxyphenol		+	6.24	341.138	137.059	0.003007981	0.003432651	0.004160658	0.003700904
111	4-Phenyl-2-butanol	Benzene and substituted derivatives	+	5.82	151.111	133.101	5.34041E-05	5.41893E-05	4.73797E-05	3.47817E-05
112	4-Hydroxy-6,9-dimethyl-3-methylene-3a,5,6,6a,7,9b-hexahydroazuleno[4,5-b]furan-2,8(3H,4H)-dione		+	6.32	263.127	245.116	1.92687E-05	2.02623E-05	2.2741E-05	2.12644E-05
113	4-hydroxy-3-(3-methylbut-2-en-1-yl)benzoic acid	Benzene and substituted derivatives	+	6.29	207.101	151.039	0.015842083	0.018954614	0.018498658	0.011528827
114	4-Aminocatechol		+	0.98	126.055	53.039	4.49621E-05	4.7888E-05	5.39564E-05	5.01086E-05
115	4-[5-[[4-[5-[acetyl(hydroxy)amino]pentylamino]-4-oxobutanoyl]-hydroxyamino]pentylamino]-4-oxobutanoic acid		+	9.72	478.293	337.272	0.034311036	0.033678328	0.037955165	0.049172572
116	4-(3-Pyridyl)-3-butenoic acid	Pyridines and derivatives	+	4.34	164.07	146.06	0.001370572	0.001767765	0.001827027	0.001376046
117	4-(2-Hydroxyethyl)phenyl beta-D-glucopyranoside		+	4.69	318.155	85.028	2.17635E-05	1.65788E-05	2.46626E-05	1.63996E-05
118	3-p-Coumaroylquinic acid		-	5.24	337.093	163.04	0.004306173	0.005267377	0.004278262	0.002463677

119	3-O-p-Coumaroylquinic acid	Organooxygen compounds	+	6.2	339.101	147.044	3.09732E-05	3.00077E-05	4.1583E-05	2.12351E-05
120	3-Methyl-cis,cis-hexadienedioate		+	0.99	157.049	95.049	0.000104313	0.000107972	0.000105923	0.000122963
121	3-Methyl-1-phenyl-3-pentanol	Benzene and substituted derivatives	+	6.65	179.142	161.132	0.000514764	0.000512848	0.000634282	0.000392365
122	3-Methyl-1-(2,4,6-trihydroxyphenyl)-1-butanone	Benzene and substituted derivatives	+	6.67	211.096	155.033	0.000714973	0.000240931	0.000425754	0.000331379
123	3-METHOXYCATECHOL		+	1	141.054	53.039	0.000168203	0.000179274	0.000199181	0.000174761
124	3-Hydroxy-1-(3-hydroxy-4-methoxyphenyl)-2-{4-[(1E)-3-hydroxy-1-propen-1-yl]-2-methoxyphenoxy}propyl beta-D-glucopyranoside		+	5.47	521.201	131.049	0.001205182	0.00142389	0.00161588	0.002242511
125	3-Buten-1-amine		+	0.72	72.081	55.054	5.83093E-05	6.42468E-05	6.57483E-05	5.95065E-05
126	3'-AMP	Ribonucleoside 3'-phosphates	+	1.01	348.069	136.061	0.012007882	0.011394057	0.012765108	0.013530061
127	3-[2-(4-Hydroxy-3-methoxyphenyl)-3-(hydroxymethyl)-7-methoxy-2,3-dihydro-1-benzofuran-5-yl]propyl beta-D-glucopyranoside		+	5.98	540.244	331.153	0.000919613	0.001044911	0.00119615	0.001238601
128	3,7-Bisaboladiene-2,8-dione	Prenol lipids	+	7.37	235.168	57.07	9.67706E-05	0.000106073	0.000147431	6.22639E-05
129	3,5-Dimethylhexanal		+	0.71	129.127	69.033	0.001051701	0.001216847	0.001357999	0.00117833
130	2-Chlorobenzoate		-	2.05	154.998	111.009	2.26442E-06	3.00528E-06	4.06706E-06	1.81348E-06
131	2-Butenoic acid, 2-methyl-, (3aR,4R,5R,9aS,9bR)-2,3,3a,4,5,7,9a,9b-octahydro-4-hydroxy-6,9-dimethyl-3-methylene-2,7-dioxoazuleno[4,5-b]furan-5-yl ester, (2Z)-		+	7.36	341.138	323.126	1.80388E-05	2.11925E-05	1.98003E-05	3.13005E-05
132	2-Butanone, 4-[3-(beta-D-glucopyranosyloxy)-4-hydroxy-2,6,6-trimethyl-1-cyclohexen-1-yl]-		+	5.38	389.216	209.153	0.000107217	9.97494E-05	0.000121777	9.76819E-05
133	2-[3-[4-[1,3-dihydroxy-1-(4-hydroxy-3-methoxyphenyl)propan-2-yl]oxy-3-methoxyphenyl]propoxy]-6-(hydroxymethyl)oxane-3,4,5-triol		+	5.53	558.254	331.153	0.000464286	0.00055	0.00063	0.00063

								1508	283	1375
134	2,2,6,7-Tetramethylbicyclo[4.3.0]nona-1(9),4-diene-7,8-diol	Organooxygen compounds	+	5.76	209.153	121.101	0.000612395	0.00060 6151	0.00074 2534	0.00067 0593
135	2-(hydroxymethyl)-6-[4-[(2S,3S)-3-(hydroxymethyl)-5-[(E)-3-hydroxyprop-1-en yl]-7-methoxy-2,3-dihydro-1-benzofuran-2-yl]-2-methoxyphenoxy]oxane-3,4,5-t riol		+	5.98	503.191	137.059	0.000553875	0.00052 8721	0.00064 0523	0.00104 8369
136	2-(6-hydroxy-6-methylheptyl)-2H-furan-5-one		+	6.49	195.137	121.101	0.000250485	0.00021 8807	0.00031 3962	0.00016 3652
137	2-(4-Methylphenyl)propanal	Prenol lipids	+	5.62	149.096	91.054	0.005552818	0.00598 3132	0.00680 1664	0.00444 2392
138	2(3H)-Furanone, dihydro-3-methylene-4-[(2E)-3-methyl-4-(tetrahydro-4-methyl-5-oxo-2-furanyl) -2-buten-1-yl]-, (4R)-		+	6.29	265.142	105.07	0.001101356	0.00108 2287	0.00160 199	0.00104 2768
139	1-Phenanthrenecarboxylic acid, 1,2,3,4,4a,9,10,10a-octahydro-1,4a-dimethyl-7-(1-methylethyl)-9-oxo-		+	10.2	315.195	187.111	0.000280074	0.00025 4872	0.00034 6269	0.00028 3989
140	1H-Indene-3-carboxylic acid, 3a,4,5,6,7,7a-hexahydro-3a,7,7-trimethyl-		+	6.47	209.153	191.142	0.000631833	0.00069 5597	0.00076 8887	0.00058 9649
141	1-deoxy-1-(N6-lysino)-D-fructose	Carboxylic acids and derivatives	-	0.85	132.03	88.04	0.000124711	8.88025 E-05	0.00012 2143	8.48828 E-05
142	1-AMINOCYCLOBUTANE CARBOXYLIC ACID		+	4.54	116.07	70.065	0.000639231	0.00081 6785	0.00088 072	0.00072 8186
143	1,2-Epoxy-p-menth-8-ene	Oxepanes	+	5.92	153.127	135.117	1.80996E-05	2.11083 E-05	1.92466 E-05	1.866E-0 5
144	[4-acetyloxy-2,5-dihydroxy-6-(hydroxymethyl)oxan-3-yl] (E)-3-(4-hydroxyphenyl)prop-2-enoate		+	7.03	351.107	147.044	0.01901877	0.01950 0881	0.01635 3135	0.02403 9873
145	(S)-Abscisic acid	Prenol lipids	-	7.13	263.129	219.139	1.77723E-05	4.42114 E-05	3.05304 E-05	2.70902 E-05
146	(E)-hex-2-enedioic acid		+	14.52	145.049	99.044	2.14989E-06	2.39294 E-06	2.823E-0 6	4.10672 E-06
147	(E)-2-Methylglutaconic acid	Fatty Acyls	+	0.99	145.049	99.044	3.66203E-05	3.40005	3.63792	3.34211

								E-05	E-05	E-05
148	(E)-2,6-Dimethyl-2,5-heptadienoic acid	Fatty Acyls	+	6.13	155.106	109.101	9.16245E-05	0.00010439	0.000110256	8.12279E-05
149	(Â±)-Tryptophan	Indoles and derivatives	+	5.03	205.096	188.07	5.58486E-05	5.33469E-05	7.19317E-05	6.53974E-05
150	(Â±)-4-Methylene-2-pyrrolidinecarboxylic acid	Carboxylic acids and derivatives	+	1.01	128.07	55.054	0.001453904	0.00160278	0.001656592	0.001663743
151	(9E)-11a-Hydroxy-3,6,10-trimethyl-7,8,11,11a-tetrahydrocyclodeca[b]furan-2,5(4H,6H)-dione		+	5.71	247.132	229.122	0.000361314	0.000517983	0.000463049	0.000515933
152	(3R,5R)-4-[(E)-3-(3,4-dihydroxyphenyl)prop-2-enoyl]oxy-1,3,5-trihydroxycyclohexane-1-carboxylic acid		+	4.94	355.102	163.038	0.01140685	0.010184155	0.012167894	0.009179451
153	(3-Hydroxy-2-oxo-2,3-dihydro-1H-indol-3-yl)acetic acid		+	4.47	208.06	146.06	0.000161638	0.000155698	0.00017392	9.90682E-05
154	(2S)-{[6-O-(beta-D-Glucopyranosyl)-beta-D-glucopyranosyl]oxy}(phenyl)acetonitrile		+	5.41	475.192	85.028	0.002148378	0.001034485	0.001035352	0.00106918
155	(1R,3R,4S,5R)-1,3,4-Trihydroxy-5-{[(2E)-3-(4-hydroxy-3-methoxyphenyl)-2-propenoyl]oxy}cyclohexanecarboxylic acid		+	5.41	369.117	177.054	0.00045608	0.000480196	0.000534904	0.00049033
156	(±)8(9)-EET methyl ester		+	9.25	335.258	81.07	0.000441338	0.000411644	0.000448152	0.000575066
157	(+)-sabinone		+	6.18	151.111	81.07	0.000520615	0.000405366	0.000591102	0.000499146
158	(+)-Epicatechin	Flavonoids	-	5.33	289.071	109.029	0.012532022	0.013903576	0.014140441	0.013505015
159	2H-Oxireno[1,10a]phenanthro[3,2-b]furan-10(11bH)-one, 5,7-bis(acetyloxy)-3,3a,4,5,6,7,7a,7b,8,8a-decahydro-4,4,7a,11-tetramethyl-, (1aS,3aR,5S,7S,7aR,7bS,8aR,11bR)-		+	9.38	415.211	119.085	0.000656839	0.000697919	0.000984615	0.00071857

Table S2

Number	Compound name	Class
1	α-Linolenoyl Ethanolamide	
2	Valsartan acid	
3	Triphenylphosphate	
4	Thymoquinone	Organooxygen compounds
5	Threonic acid	Organooxygen compounds
6	Sweroside	
7	Sucrose	Organooxygen compounds
8	Salicylic acid	Benzene and substituted derivatives
9	Quinic acid	Organooxygen compounds
10	Pyrrolidonecarboxylic acid	Carboxylic acids and derivatives
11	p-Xylene	Benzene and substituted derivatives
12	Pseudoionone	Prenol lipids
13	p-Mentha-1,3,8-triene	Prenol lipids
14	p-Mentha-1,3,5,8-tetraene	Benzene and substituted derivatives
15	Perillyl acetate	Prenol lipids
16	PC(16:0/0:0)[U] / PC(16:0/0:0)[rac]	
17	Pantothenic acid	Alcohols and polyols
18	Palmitic amide	Fatty Acyls
19	o-Ethyltoluene	Benzene and substituted derivatives
20	N-Oleoylethanolamine	Organonitrogen compounds
21	Niacinamide	Pyridines and derivatives
22	NCGC00381407-01	
23	Methyl cinnamate	Cinnamic acids and derivatives
24	Malic acid	Hydroxy acids and derivatives
25	LysoPE(16:0/0:0)	Glycerophospholipids

26	L-beta-Homothreonine	
27	Iridotrial	
28	Hymecromone methyl ether	
29	Glycerophosphocholine	Glycerophospholipids
30	Galactaric acid	Organooxygen compounds
31	Egomaketone	Carbonyl compounds
32	DL-Glutamate	Carboxylic acids and derivatives
33	D-Glucuronic acid	Organooxygen compounds
34	Cytosine	Diazines
35	Cytidine	Pyrimidine nucleosides
36	Citrulline	Carboxylic acids and derivatives
37	Citral propylene glycol acetal	Dioxolanes
38	Chlorogenic acid	Organooxygen compounds
39	Biliverdin	Tetrapyrroles and derivatives
40	beta-D-Glucopyranoside, 4-(3-hydroxypropyl)-2-methoxyphenyl	
41	beta-D-Glucopyranose, 1-O-[(2E,6E)-8-hydroxy-2,6-dimethyl-1-oxo-2,6-octadien-1-yl]-	
42	6,10-Dimethyl-5(E),9-undecadien-2-one	
43	5'-Methylthioadenosine	5'-deoxyribonucleosides
44	4-hydroxy-3-(3-methylbut-2-en-1-yl)benzoic acid	Benzene and substituted derivatives
45	4-[5-[[4-[5-[acetyl(hydroxy)amino]pentylamino]-4-oxobutanoyl]-hydroxyamino]pentylamino]-4-oxobutanoic acid	
46	4-(2-Hydroxyethyl)phenyl beta-D-glucopyranoside	
47	3-p-Coumaroylquinic acid	
48	3-O-p-Coumaroylquinic acid	Organooxygen compounds
49	3-Methyl-1-phenyl-3-pentanol	Benzene and substituted derivatives
50	3,7-Bisaboladiene-2,8-dione	Prenol lipids
51	3,5-Dimethylhexanal	
52	2-Chlorobenzoate	
53	2-Butenoic acid, 2-methyl-, (3aR,4R,5R,9aS,9bR)-2,3,3a,4,5,7,9a,9b-octahydro-4-hydroxy-6,9-dimethyl-3-methylene-2,7-dioxoazuleno[4,5-b]furan-5-yl ester, (2Z)-	

54	2,2,6,7-Tetramethylbicyclo[4.3.0]nona-1(9),4-diene-7,8-diol	Organooxygen compounds
55	2-(hydroxymethyl)-6-[4-[(2S,3S)-3-(hydroxymethyl)-5-[(E)-3-hydroxyprop-1-enyl]-7-methoxy-2,3-dihydro-1-benzofuran-2-yl]-2-methoxyphenoxy]oxane-3,4,5-triol	
56	2-(6-hydroxy-6-methylheptyl)-2H-furan-5-one	
57	2-(4-Methylphenyl)propanal	Prenol lipids
58	2(3H)-Furanone, dihydro-3-methylene-4-[(2E)-3-methyl-4-(tetrahydro-4-methyl-5-oxo-2-furanyl)-2-buten-1-yl]-, (4R)-	
59	1-Phenanthrenecarboxylic acid, 1,2,3,4,4a,9,10,10a-octahydro-1,4a-dimethyl-7-(1-methylethyl)-9-oxo-	
60	(E)-2,6-Dimethyl-2,5-heptadienoic acid	Fatty Acyls
61	(+)-sabinone	
62	2H-Oxireno[1,10a]phenanthro[3,2-b]furan-10(11bH)-one, 5,7-bis(acetyloxy)-3,3a,4,5,6,7,7a,7b,8,8a-decahydro-4,4,7a,11-tetramethyl-, (1aS,3aR,5S,7S,7aR,7bS,8aR,11bR)-	
