# Supplementary material for: Discrimination of Geographical Origin of Agricultural Products From Small-Scale Districts by Widely Targeted Metabolomics With a Case Study on Pinggu Peach
Source: Front Nutr. 2022 May 24;9:891302. doi: 10.3389/fnut.2022.891302 (PMC9172448; doi:10.3389/fnut.2022.891302)
Supplement: Supplementary file 2 [file Data_Sheet_2.PDF]

# Analytical Greenness report sheet

13/04/2022 15:36:51

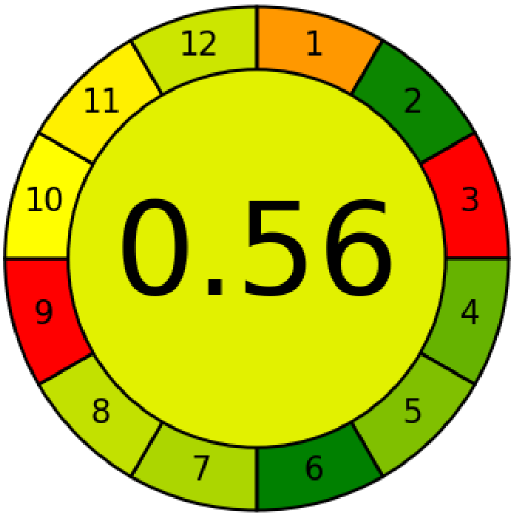


| Criteria | Score | Weight |
|---|---|---|
| 1. Direct analytical techniques should be applied to avoid sample treatment. | 0.3 | 2 |
| 2. Minimal sample size and minimal number of samples are goals. | 0.98 | 2 |
| 3. If possible, measurements should be performed in situ. | 0.0 | 2 |
| 4. Integration of analytical processes and operations saves energy and reduces the use of reagents. | 0.8 | 2 |
| 5. Automated and miniaturized methods should be selected. | 0.75 | 2 |
| 6. Derivatization should be avoided. | 1.0 | 2 |
| 7. Generation of a large volume of analytical waste should be avoided, and proper management of analytical waste should be provided. | 0.66 | 2 |
| 8. Multi-analyte or multi-parameter methods are preferred versus methods using one analyte at a time. | 0.62 | 2 |
| 9. The use of energy should be minimized. | 0.0 | 2 |
| 10. Reagents obtained from renewable sources should be preferred. | 0.5 | 2 |
| 11. Toxic reagents should be eliminated or replaced. | 0.47 | 2 |
| 12. Operator's safety should be increased. | 0.6 | 2 |
